# Using Creative Dance to Promote Autonomy Development in Young Children in China: An Intervention Study

**DOI:** 10.3390/bs15111492

**Published:** 2025-11-03

**Authors:** Xin Lin, Chan Zhou, Longqi Yu, Xinyue Zhang, Xiaofan Cao, Chenyang Guan

**Affiliations:** 1 School of Art, Beijing Sport University, Beijing 100084, China; 15070317040@163.com (X.L.); 13381219536@163.com (X.Z.); 2 School of Education, Beijing Sport University, Beijing 100084, China; zhouchanxj@163.com; 3 The School of International Education and Exchange, Beijing Sport University, Beijing 100084, China; caoxiaofan@bsu.edu.cn; 4 School of Leisure and Society, Capital University of Physical Education and Sports, Beijing 100191, China; guanchenyang@sdtyxy6.wecom.work

**Keywords:** autonomy, Creative Dance, autonomy need, children

## Abstract

Introduction: Creative Dance, as an educational approach to physical activity emphasizing autonomous exploration and creative expression, has demonstrated significant benefits for children’s cognitive development and independent learning. This study aimed to examine the effects of Creative Dance on the development of autonomy among Chinese children aged 4 to 6 years, and to investigate the mediating role of autonomy need satisfaction in this process. Methods: A randomized, single-blind, two-arm experimental design was utilized, with classrooms serving as the unit of assignment. A total of 102 children aged 4–6 years were randomly allocated to either an experimental group (Creative Dance) or a control group (DanceSport). The children’s autonomy was measured pre- and post-intervention using parent-proxy reports. In addition, qualitative interviews and video observations were conducted to assess the degree of autonomy need satisfaction experienced by the children during the Creative Dance intervention. Results: Analysis of the pre-intervention data revealed no statistically significant differences in autonomy scores between the experimental and control groups. Post-intervention, the children in the experimental group scored significantly higher across all dimensions of autonomy compared to those in the control group. Mediation analysis indicated that autonomy need satisfaction significantly mediated the effect of Creative Dance on self-assertion, but not on self-reliance or self-control. Discussion: Creative Dance constitutes an effective intervention for supporting autonomy need satisfaction and promoting overall autonomy development in young children. Satisfaction of autonomy needs serves as a key mechanism through which Creative Dance enhances self-assertion. These findings provide empirical evidence supporting the incorporation of Creative Dance into early childhood education as a means of promoting physical and mental development.

## 1. Introduction

Autonomy can be conceptualized as an individual’s ability to pursue and realize personally endorsed goals and aspirations through deliberate effort (Zou, 2006). Children’s autonomy is typically understood to encompass three fundamental facets: self-assertion, self-control, and self-reliance. Extensive empirical literature underscores the significance of autonomy as a foundational component of children’s personality formation and social development, with evidence indicating its substantial influence on psychological well-being, future adaptability, and the maturation of social competencies (Deci & Ryan, 2000). Conversely, a lack of support for children’s autonomy—or, more detrimentally, actively undermining it through controlling behaviors—has been linked to heightened levels of anxiety among children (Gao et al., 2016). This is particularly salient for preschoolers, a stage at which autonomy support proves pivotal in fostering a healthy sense of self and, ultimately, self-regulation (Ke, 2023). Thus, a pressing question arises: How can we effectively nurture autonomy in young children? It is noteworthy that the establishment of clear and reasonable rules and expectations does not constrain children’s autonomy; rather, it offers secure boundaries and psychological reassurance that facilitate the maturation of autonomy (Abula et al., 2020). Crucially, educational activities must be crafted with sufficient flexibility to afford young children with abundant opportunities for autonomous choice and self-expression within well-defined parameters (N. Hu & Li, 2025; Yu & Buck, 2025; Sanchez, 2025). These insights indicate that practices which harmoniously integrate structure with autonomy, and rules with openness, can most effectively cultivate autonomy in early childhood.

As a new type of physical activity education that cherishes individual creativity and autonomy, Creative Dance, which combines the qualities of structure and autonomy, rules and openness, has gained widespread attention at home and abroad in recent years. Numerous empirical studies have shown that Creative Dance significantly and positively affects young children’s motor skills, cognitive ability, and emotional regulation (Konstantinidou, 2023). In particular, the scaffolding teaching and improvisational dance in Creative Dance effectively integrate rule-based guidance and autonomy (Yu & Buck, 2024; Lv, 2024). The interaction between the two creates a favorable developmental field for young children with a clear framework for exploration and freedom of expression, thus demonstrating a unique potential for promoting young children’s autonomy. Furthermore, Piaget contended that bodily movement underpins the development of cognitive structures (Piaget & Inhelder, 1941). Preschoolers in the preoperational stage, despite emerging language, rely heavily on concrete actions, manifesting a mode of “thinking through the body” (Bayanova et al., 2022). Creative Dance, as an educational activity, aligns closely with the developmental characteristics of young children, promoting comprehensive advancement in the cognitive, emotional, and social domains while affording them varied and autonomous opportunities for exploration (Carson et al., 2016; Yuan et al., 2025).

Basic Psychological Needs Theory (BPNT) provides a solid theoretical foundation for understanding the causal mechanism through which Creative Dance promotes the development of young children’s autonomy by satisfying their fundamental need for autonomy. The key concept in BPNT is the perception of and desire for freedom and ownership over one’s behaviors (Kashdan et al., 2004). Research indicates that when children’s need for autonomy is supported and satisfied in both their family and educational environments—for example, by offering choices, valuing their perspectives, and explaining the rationale behind rules—their autonomy is strengthened, leading to sustained self-development (Ryan & Deci, 2017). An analysis of the existing literature reveals that the activity design of Creative Dance is highly aligned with the key elements required to satisfy this need. For instance, its pedagogical focus on elements rather than structured combinations, its provision of improvisational space for self-directed exploration, and its teacher-as-facilitator approach all strongly align with supporting young children’s autonomous learning (Tsompanaki & Magos, 2022).

However, although prior studies have laid a foundation for understanding the relationships among Creative Dance, autonomy, and the need for autonomy, systematic empirical verification of these relationships within preschool educational practice remains limited. Given that Basic Psychological Needs Theory (BPNT) offers a comprehensive framework for elucidating this complex relationship, this study employed the BPNT framework to systematically examine the causal mechanism through which Creative Dance fosters young children’s autonomy development. Specifically, through a longitudinal intervention study, we aimed to investigate the effect of Creative Dance on the development of autonomy in young children and to empirically test the mediating role of autonomy need satisfaction, thereby providing empirical evidence for the applicability of BPNT in preschool education and enriching the literature on autonomy and Creative Dance education.

## 2. Literature Review

### 2.1. Autonomy

Autonomy is typically defined as an individual’s willingness and capacity to make choices and decisions, and to take actions independently in accordance with a personally endorsed and coherent system of values or goals (Ke, 2023). Throughout early socialization and personality development, autonomy plays a pivotal role and constitutes a core component in the formation of self-concept. In the Chinese literature, Zou divided autonomy into three dimensions: self-reliance, which refers to the capacity to complete tasks by drawing on one’s own resources and to refrain, when appropriate, from seeking or depending on help from others; self-control, which refers to the effective regulation of one’s behavior and emotions and the inhibition of unreasonable impulses or desires in order to align with goals or social norms; and self-assertion, which refers to the ability to clearly and appropriately express one’s needs, viewpoints, and feelings in interpersonal contexts and to make relatively independent decisions free from unreasonable domination (Zou, 2006). With respect to young children, the research indicates that purposeful, planned, and systematic educational activities can effectively promote and positively shape the development of autonomy (Su & Reeve, 2011).

There is empirical evidence showing that the two key elements that shape young children’s autonomy development are a relaxed and open educational environment and a balance between freedom and rules. First, a relaxed and open educational environment is a prerequisite for cultivating autonomy. In low-pressure classroom settings, children’s anxiety and psychological distance are markedly reduced, making them more willing to proactively explore new activities, thereby facilitating autonomy development (Flunger et al., 2019). Specific strategies include granting children choice over activities, control over materials, and agency in time allocation while providing ample space for play and social interaction. This sense of freedom alleviates psychological stress and energizes intrinsic motivation, which in turn promotes autonomy development (Koteva-Mojsovska & Nikodinovska Banchotovska, 2019). Second, balancing freedom with structure (i.e., rules) is a critical strategy for fostering autonomy (Bao & Lam, 2008). Traditional educational perspectives have expressed concerns that structure and rules may suppress children’s autonomy and creativity. However, recent empirical research indicates that clear, reasonable, and predictable rules are not obstacles; rather, explicit rules and behavioral expectations provide necessary safety boundaries and a psychological support system (Javier et al., 2022). Appropriate rules delineate the scope of “what can be done” and clarify the pathways for “how to do it,” enabling children to exercise autonomy more confidently and proactively within an established framework and gradually develop independence. Therefore, when designing activities, educators should strive for a dynamic balance between freedom and rules, maximizing opportunities for autonomous choice, individualized expression, and independent problem solving within a supportive structure.

### 2.2. Creative Dance

As a pedagogy that emphasizes listening to the body and attending to internal sensations, the aims of Creative Dance education are to guide participants to focus on their emotional experiences, enhance the quality of interactions with others and the surrounding environment, and develop body awareness as well as movement knowledge and skills, ultimately promoting holistic physical and mental health (Tang & Huang, 2023). Creative dance, through the continuous exploration of Chinese and international dance educators such as Laban, Doubler, Barbara, Bang, and Lv, has evolved over nearly a century to assume diverse forms globally, deeply integrating with various cultures (Doubler, 1940; Laban, 1948; Mettler, 1960; Bang, 1994; Huo, 2015). In China, the “Quality-Oriented Education Dance” (QOED) system developed by Lv Yisheng’s team marks a new era in the evolution of creative dance (He & Wang, 2024). The core of this curriculum lies in stimulating students’ intrinsic motivation through a highly integrated, multidimensional teaching and learning approach, enabling them to consciously cultivate the qualities they need (Lv, 2016). At the compulsory education level in China, Creative Dance is primarily implemented within the dance strand of the arts curriculum and is taught by arts (dance) teachers. Additionally, some schools offer it as a supplementary component in physical education classes and extracurricular clubs, aiming to stimulate students’ creativity, emotional expression, and body awareness. In kindergarten and university settings, Creative Dance is mainly integrated into daily creative activities or offered as general education arts electives, emphasizing holistic development and aesthetic experience.

A large number of studies have suggested that Creative Dance holds considerable potential in promoting young children’s autonomy development (Biasutti & Habe, 2021). First, Creative Dance emphasizes respect for bodily autonomy: rather than requiring students to execute pre-specified standard movements, it encourages them to explore bodily capacities and self-potential through dance (Cherrière et al., 2020). This affirmation and acceptance of children’s autonomous expression help cultivate an open, relaxed, and safe exploratory climate—conditions that are crucial for autonomy development. Second, the effective integration of scaffolded teaching with improvisational dance enables an organic fusion of rule-guided and autonomous exploration (Engdahl et al., 2021). Specifically, scaffolding underscores the provision of timely, appropriately calibrated support and guidance from adults or teachers, contingent on children’s developmental level or learning status, thereby helping them explore within clear and safe boundaries. Improvisational dance, in turn, affords space for free expression and active creation, encouraging the development of individuality and creativity in an open environment (Larsson et al., 2025). Taken together, the distinctive educational philosophy of Creative Dance—especially its respect for bodily autonomy and the synergistic interplay between structured guidance and improvisation—constitutes a pedagogical framework that combines systematic support with free exploration. This suggests that Creative Dance is a potent vehicle for fostering young children’s autonomy.

### 2.3. Basic Psychological Needs Theory

Within the framework of Self-Determination Theory (SDT), Basic Psychological Needs Theory (BPNT) is recognized as a central mini-theory.

BPNT posits that there are three fundamental and universally applicable psychological needs—autonomy, competence, and relatedness—which are essential for optimal human functioning and well-being.

The need for autonomy refers to the experience of engaging in behaviors that are self-chosen and volitional rather than being guided by external coercion, control, or pressure (Deci & Ryan, 2000).

As necessary conditions for the growth and integration of personality and cognitive structures, these basic psychological needs motivate individuals to seek their fulfillment. When individuals perceive freedom of choice and a sense of volition within their environment, their need for autonomy is effectively strengthened (Javier et al., 2022). Research indicates that educational environments that support autonomy and provide choices satisfy the need for autonomy as they grant learners autonomy while offering appropriate guidance rather than external control (Niemiec & Ryan, 2009). The pedagogical philosophy and instructional routines of Creative Dance have been found to facilitate the satisfaction of students’ need for autonomy (Goulimaris et al., 2014). On the one hand, Creative Dance is student-centered: its instructional aim is to unlock children’s potential through bodily movement rather than merely mastering fixed dance combinations. In Creative Dance classes, the teachers’ role is not as a director but a facilitator—for example, by taking into account the students’ perspectives, providing meaningful rationales, and offering options—which enhances the students’ perceived autonomy and thereby satisfies their need for autonomy (Engdahl et al., 2021). On the other hand, open-ended sessions—such as self-selected partners and improvisational dance—grant children the right to make independent choices and to express themselves freely, enabling them to translate their intentions, ideas, and unique feelings into action (Alper & Ulutaş, 2022). Such autonomy-supportive instructional design continually satisfies children’s need for autonomy and promotes the internalization of autonomy into their everyday volitional behaviors, laying the foundation for deeper motivation and positive development (Chen, 2001).

In summary, although the synthesis of the existing literature suggested that Creative Dance may positively affect young children’s autonomy development, specific verification through empirical research remains lacking. Meanwhile, the need for autonomy has also provided an important theoretical framework for understanding the causal mechanism underlying how Creative Dance promotes the development of young children’s autonomy. Against this backdrop and building on the foundations and limitations of previous studies, this study aimed to answer the following research questions:

RQ1: As a form of physical activity-based education that emphasizes participants’ independent exploration and creative expression, does Creative Dance promote the development of young children’s autonomy?

RQ2: Does the satisfaction of young children’s need for autonomy play a mediating role in the process through which Creative Dance promotes the development of young children’s autonomy?

## 3. Materials and Methods

### 3.1. Participants

Before commencing participant recruitment, a priori power analysis was performed using G*Power 3.1.9.7 (Faul et al., 2007) to determine the necessary sample size for detecting a possible interaction effect using repeated-measures ANOVA of two groups. The calculation utilized a two-tailed test with an alpha level of 0.05 and targeting an effect size of 0.25 and statistical power of 0.95. The power analysis indicated that at least 54 participants—27 in each group—would be needed to ensure adequate sensitivity and reliability of the statistical inference. Accordingly, 116 children were initially invited to take part in the study. After applying stringent eligibility criteria, 14 children were excluded, resulting in a final sample of 102 participants (52 in the experimental group and 50 in the control group). The final cohort comprised 46 boys and 56 girls, thereby fulfilling the calculated requirements for sample size and ensuring the robustness of subsequent analyses.

The inclusion criteria for the participants in this study were as follows: (1) Aged 4–6 years and capable of participating in the experiment; (2) Children voluntarily participated, and their parents signed the informed consent form; (3) Having no severe cognitive impairments or mental illnesses (initially screened by the class advisor). The exclusion criteria were: (1) Participation in other dance activities during the experimental intervention period; (2) Failure to complete the experiment as required, with an attendance rate lower than 60%; (3) Failure to fully participate in the pre- and post- intervention phases of the teaching experiment.

### 3.2. Measures

#### 3.2.1. Children’s Autonomy Scale

Chinese scholar Zou Xiaoyan developed a scale for measuring the autonomy of preschoolers using reports from parent proxies (Yang & Dong, 2005). This scale measures several aspects of autonomy:

Self-Reliance: The reliance on one’s own strength and the tendency to relatively infrequently seek help from others. The opposite aspect is dependence.

Self-Control: The ability to actively restrain one’s unreasonable desires and regulate one’s behavior. The opposite aspect is capriciousness.

Self-Assertion: The ability to make decisions on one’s own, free from the influence and domination of others.

The questionnaire consists of 20 questions divided into three dimensions: 6 questions on self-reliance, 6 on self-assertion, and 8 on self-control. The instrument utilizes a 5-point Likert scale ranging from 1 to 5, where a score of “1” indicates that the described situation has never occurred for the child, while a score of “5” indicates that it occurs consistently. Higher aggregate scores are indicative of greater levels of autonomy in children. It is important to note that items 7, 8, and 16 are reverse-scored. In the present study, Cronbach’s alpha coefficients for both the overall scale and its subscales exceeded 0.70, demonstrating satisfactory internal consistency and the reliability of the measurement tool.

#### 3.2.2. The Need for Autonomy Scale

To assess autonomy need satisfaction, this study utilized the autonomy dimension subscale from the Chinese version of the Basic Psychological Needs Scale for the Classroom, which is applicable to various physical activity programs and has been translated and validated by Hu Xiaoqing and colleagues (X. Q. Hu, 2022). Given young children’s developmental limitations in written language comprehension and attention span, the items were adapted for administration through a structured interview format. The children responded to each item using a 4-point Likert-type scale, which was illustrated as a ladder diagram (1 = almost never, 2 = sometimes, 3 = frequently, and 4 = always). For example, “I feel that the activities I do in the classroom are like my own choices and decisions.”

#### 3.2.3. Observation of Autonomous Need-Supportive Teaching Behaviors

To further enhance the validity of the assessment regarding young children’s perceptions of need—supportive teaching, this study incorporated the Observation of Autonomous Need-Supportive Teaching Behaviors subscale from the List of Observed Need-Supportive Teaching Behaviors developed by Haerens et al. This subscale was used to complement the indicators reflecting the children’s experiences of autonomy within the study context. These instructional behaviors were systematically monitored and recorded at five-minute intervals using a 4-point observational frequency scale (0 = never observed, 1 = sometimes observed, 2 = often observed, and 3 = always observed; Haerens et al., 2013). The reliability analysis yielded a Cronbach’s alpha coefficient greater than 0.70, indicating satisfactory internal consistency and the psychometric robustness of this instrument.

The interviews to assess the children’s autonomy needs could not be tested for reliability due to it consisting of a single question. To ensure the reliability of the research data, this study incorporated autonomy-needs-supportive classroom observation as a complementary way of examining the children’s autonomy needs. Specifically, we video-recorded all the lessons during the experiment and randomly selected 50% of the classroom videos for analysis using the Autonomous Needs Supportive Teaching Behavior Observation Tool at the end of the lessons. Two coders with more than 3 years of experience teaching dance were invited to conduct the coding (the coders were unaware of the purpose of the study and the experimental groupings). To ensure coding reliability, the coders received systematic training. They practiced pre-coding until the two coders achieved a coding consistency greater than 95% before they started to code the experimental video data. Kappa analysis of the coded data from the classroom observations of autonomous needs-supportive teaching showed a Kappa value of 0.87, indicating good video-coding consistency the teachers’ autonomous needs-supportive teaching behaviors. We then conducted correlational analyses between need-for-autonomy scores and classroom observations of autonomy-supportive teaching. This provided additional validity evidence for the need-for-autonomy measure. The results indicated a significant correlation between the two variables (*r* = 0.665, *p* < 0.001, *n* = 102). In summary, the test instruments used in this study all had high reliability and were able to provide a solid measurement basis for the findings.

### 3.3. Intervention Program

The design of the Creative Dance intervention program in this study was based on Laban Movement Analysis (LMA), which divides body movement into four parts (BESS): Body, Effort, Shape, and Space, which cover 15 movement elements (Groff, 1995). This study referred to the theoretical and practical frameworks and educational suggestions (including the sequence of movement elements, degree of difficulty, teaching methods, etc.) put forward by Anne Green Gilbert, a pioneer of Creative Dance in the U.S., in her book *Creative Dance for All Ages*, and adjusted them to account for young children’s physical and mental developmental stages, with a focus on autonomous development and knowledge receptivity, to design a curriculum that is suitable for young children (Gilbert, 2015). A 10-week Creative Dance intervention program was designed (Table A1). The core objective of the Creative Dance intervention is to integrate interest-based themes with Laban movement elements to guide young children toward autonomous exploration and expression while employing autonomy-supportive instruction to satisfy their need for autonomy. In doing so, the program systematically cultivates children’s self-reliance, self-control, and self-assertion. For example, in Week 2’s “Bubbles and Jellyfish” theme, familiar, interest-based themes are introduced and threaded throughout the session to enhance engagement. In addition, the children are invited to autonomously select their movement patterns (e.g., “small bubbles” vs. “big jellyfish”) and effort level (e.g., the sustained swimming of jellyfish vs. the sudden popping of bubbles), thereby advancing the core goal of fostering autonomy. The program focuses on teaching and exploring the 13 movement elements of position, size, level, and direction, with the last two weeks of the program focusing on using the integrated elements and guiding the children to engage in integrated creative expression.

We selected DanceSport to compare to the Creative Dance intervention. This intervention was also based on the children’s physical and mental developmental stages and their knowledge acceptance, with reference to the early childhood teaching materials of the Chinese DanceSport Federation. The primary part of the 10-week intervention consisted of the cha-cha-cha as the main content of the program (Table A2). We selected DanceSport based on two primary considerations. First, Creative Dance and DanceSport are both dance-based physical activities that use bodily movements as the vehicle for education and learning. This similarity helps minimize confounding factors due to differences in activity modality that might otherwise affect young children’s autonomy development. Thus, the between-group differences can be more clearly attributed to distinctive mechanisms related to the teacher guidance style, activity format, and other features of the task content. The second reason was that the instructional mode of DanceSport stands in sharp contrast to that of Creative Dance. DanceSport emphasizes highly structured, standardized, routine-based instruction, whereas Creative Dance prioritizes unstructured, self-directed exploration and expression. Using this control intervention not only reduces the interference from activity-type differences but also enables a systematic comparison of structured versus unstructured instruction in terms of their effects on young children’s autonomy development, thereby highlighting the unique facilitative role of Creative Dance.

### 3.4. Study Procedure

This study was conducted in accordance with the Declaration of Helsinki and received approval from the Ethics Committee of Beijing Sport University (No. 2024290H). The research was carried out over a period of 12 weeks in two kindergartens located in northern China. The study procedure consisted of three phases: a pre-intervention phase (1 week), an intervention implementation phase (10 weeks), and a post-intervention phase (1 week).

This study employed a randomized, single-blind, two-arm experimental design. Children who met the inclusion criteria were randomly assigned to either the experimental group (Creative Dance) or the control group (DanceSport). The participants were blinded to their group allocation, whereas the researchers were aware of the specific intervention content provided to each group. The interventions were delivered in a classroom setting. Before the intervention, all the parents were invited to fill out the self-administered basic information questionnaire and the children’s autonomy scale at the school. The data were analyzed based on the children’s demographics and tested for homogeneity; there was no significant difference in the level of autonomy development between the two groups of children. The 10-week, 2-times-a-week, 40 min formal intervention experiment was conducted in the experimental group using the Creative Dance Intervention Program during the children’s extracurricular activity time. The control group simultaneously participated in the physical dance intervention (with the same weekly frequency and duration as the experimental group). During the experiment, all the activities were videotaped as a supplement to the subsequent interviews concerning the children’s autonomy needs fulfillment. At the end of the intervention, the parents were again invited to complete the same questionnaire given in the pre-intervention phase, and the toddlers were also required to complete an autonomous needs fulfillment interview.

### 3.5. Data Processing

In this study, the statistical analysis of the experimental data was conducted using SPSS 29.0 and R 4.5.1 software. Initially, the data were examined for normality using the Shapiro–Wilk test, alongside a homogeneity assessment through independent samples t-tests, to ensure that the prerequisites for parametric testing were satisfied. Additionally, these tests were used to confirm that the pre-intervention levels of self-reliance, self-assertion, and self-control did not significantly differ between the two groups. In the descriptive statistics, the mean and standard deviation (M ± SD) were reported to summarize each variable. To evaluate the impact of the intervention on the various dimensions of autonomy, repeated-measures ANOVA was employed. Bivariate correlation analysis was then performed to examine the relationships among the variables and to establish their suitability for mediation analysis. Subsequently, the mediating effect of need for autonomy was tested using the SPSS Process Model 4 and the Bootstrap method.

## 4. Results

To assess the normality of the autonomy data distribution prior to the formal statistical analyses, the pre- and post-intervention autonomy scores were examined using the Shapiro–Wilk test. The results indicated that significance levels for autonomy were all greater than 0.05 (*p* = 0.369 to 0.948) for both the pre- and post-intervention results, confirming that the data met the assumption of normality and were thus suitable for subsequent parametric analyses.

### 4.1. Common Method Bias

To assess the potential common method bias within the dataset, Harman’s single-factor approach was employed through an unrotated principal component analysis of all the measurement variables (Zhou & Long, 2004). The results revealed that the extraction of 14 distinct factors had eigenvalues exceeding 1, with the first factor explaining only 17.94% of the total variance—considerably lower than the frequently referenced threshold of 40%. These findings suggest that the common method bias is unlikely to pose a significant threat to the validity of the study’s results.

### 4.2. Pre-Intervention Homogeneity Test

The results (Table 1) indicated that there was no statistically significant difference in the autonomy levels of the toddlers in the control and experimental groups prior to the intervention (*p* > 0.05). Thus, the two groups were equivalent in terms of their baseline autonomy scores, permitting further comparative analyses.

### 4.3. Comparative Analysis of Autonomy Dimensions Before and After Intervention

A two-factor, two-level repeated-measures analysis of variance (ANOVA) was conducted to examine the effects of group and time on the children’s autonomy levels, with gender, age in months, and BMI included as covariates. The results are presented in Table 2.

With regard to self-reliance, a significant main effect of time was observed (*F* = 10.90, *p* < 0.001, *η*^2^ = 0.10), whereas the main effect of group was not significant (*F* = 3.53, *p* > 0.05, *η*^2^ = 0.04). Importantly, there was a significant interaction between time and group (*F* = 18.28, *p* < 0.001, *η*^2^ = 0.16), indicating that the pre- to post-intervention change in self-reliance levels differed significantly between the experimental and control groups. Simple effects analysis Table 2 showed no significant difference between groups in the pre-intervention phase (*F* = 0.23, *p* > 0.05, *η*^2^ = 0.002), whereas a significant difference was found in the post-intervention phase (*F* = 21.54, *p* < 0.001, *η*^2^ = 0.18). Within the experimental group, the self-reliance scores significantly increased from the pre- to post-intervention phase (*F* = 63.06, *p* < 0.05, *η*^2^ = 0.39), suggesting substantial intervention effects, as expected. In contrast, no significant change was found within the control group over time (*F* = 3.15, *p* > 0.05, *η*^2^ = 0.03), indicating that self-reliance remained stable in this group.

Regarding self-assertion, neither the main effect of time (*F* = 0.23, *p* > 0.05, *η*^2^ = 0.002) nor the main effect of group (*F* = 3.30, *p* > 0.05, *η*^2^ = 0.33) was significant. However, a significant interaction effect between time and group was observed (*F* = 4.55, *p* < 0.05, *η*^2^ = 0.05), suggesting that the change in self-assertion levels between the pre- and post-intervention phases differed significantly between the experimental and control groups. Simple effect analyses (Table 2) revealed no significant difference between groups at the pre-intervention phase (*F* = 0.01, *p* > 0.05, *η*^2^ = 0.000), while a significant difference emerged at the post-intervention phase (*F* = 9.47, *p* < 0.01, *η*^2^ = 0.90). Within the experimental group, the self-assertion scores significantly increased from the pre- to post-intervention phase (*F* = 23.28, *p* < 0.001, *η*^2^ = 0.19), indicating a substantial effect of the intervention, which was consistent with expectations. In contrast, the control group showed no significant change in self-assertion between the pre- and post-intervention phases (*F* = 3.00, *p* > 0.05, *η*^2^ = 0.03), indicating stability over time.

**Figure 1:**
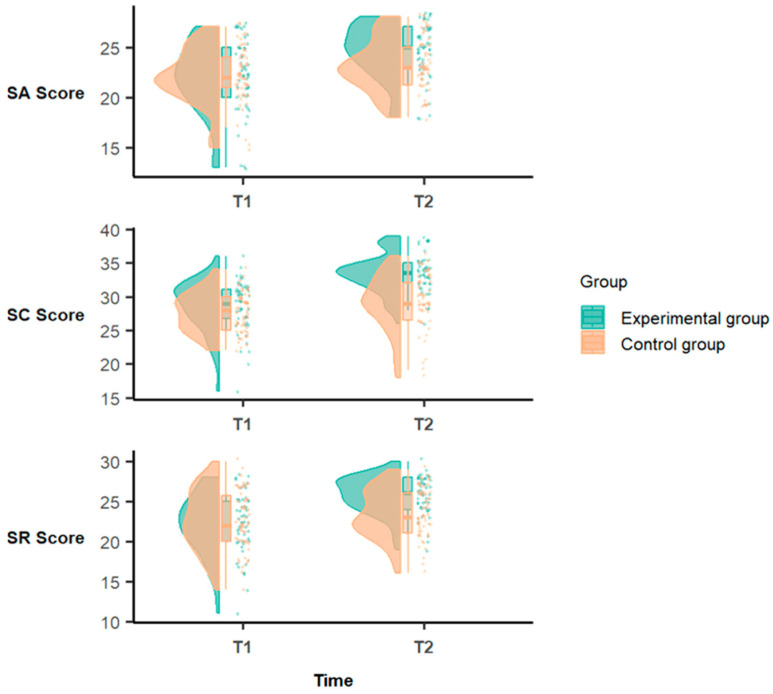
Changes in each dimension of autonomy among the children in the experimental and control groups before and after the intervention. Note: SA = self-assertion; SC = self-control; SR = self-reliance; Experimental group = Creative Dance group; Control group = DanceSport group; T1 = pre-intervention; T2 = post-intervention.

Regarding self-control, there was no significant main effect of time (*F* = 1.38, *p* > 0.05, *η*^2^ = 0.01), while a significant main effect of group was found (*F* = 24.52, *p* < 0.001, *η*^2^ = 0.20). Additionally, a significant interaction effect between time and group was observed (*F* = 19.87, *p* < 0.001, *η*^2^ = 0.17), indicating that the change in self-control levels between the pre- and post-intervention phases differed significantly between the experimental and control groups. Simple effect analyses (Table 2) revealed no significant difference between the groups in the pre-intervention phase (*F* = 3.46, *p* > 0.05, *η*^2^ = 0.03), whereas in the post-intervention phase, the difference between the groups was significant (*F* = 41.47, *p* < 0.001, *η*^2^ = 0.30). Within the experimental group, the self-control scores significantly increased from the pre- to post-intervention phase (*F* = 86.37, *p* < 0.001, *η*^2^ = 0.47), reflecting a clear impact of the intervention that was consistent with expectations. The control group also demonstrated a significant change in self-control over time (*F* = 8.09, *p* < 0.01, *η*^2^ = 0.08), which may be attributed to maturation effects or positive influences from participation in sports, dance, or interactions with the dance instructors. Notably, the increase in self-control within the experimental group was significantly greater than that observed in the control group (Figure 1).

### 4.4. Correlation Analysis

This study examined the correlations among self-reliance, self-assertion, self-control (differences in pre- and post-test scores), group and autonomy needs. The results showed pairwise significant or marginally significant positive correlations between the children’s self-reliance, self-assertion, self-control scores (differences in pre- and post-intervention scores), group, and autonomy needs (see Table 3).

### 4.5. Mediation Analysis of Autonomy Needs

This study utilized the PROCESS macro for SPSS developed by Hayes and Montoya (2017) to construct and test the mediation model. Model 4 was employed, using group as the binary independent variable (coded as “0” for the control group and “1” for the experimental group); the pre- to post-intervention phase changes in self-reliance, self-assertion, and self-control scores as the dependent variables; autonomy needs as the mediating variable; and BMI, gender, and age in months as covariates.

**Figure 2:**
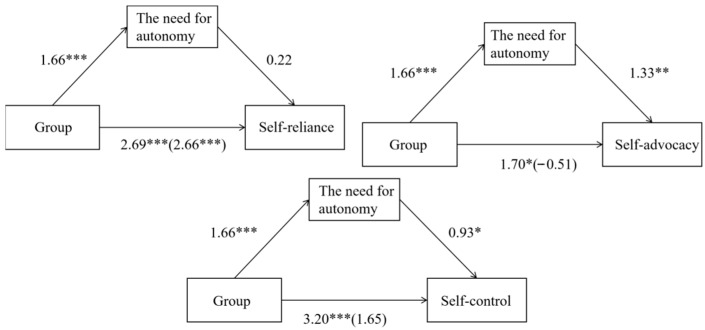
Mediating model diagram. Note: * = *p* < 0.05; ** = *p* < 0.01; *** = *p* < 0.001.

The results showed that there was a positive correlation between group and autonomy needs (*B* = 1.66, SE = 0.17, *t*(97) = 9.67, *p* < 0.001). Autonomy needs had a significant positive impact on self-assertion and self-control (self-assertion: *B* = 1.33, SE = 0.45, *t*(96) = 2.93, *p* < 0.01; self-control: *B* = 0.93, SE = 0.41, *t*(96) = 2.24, *p* < 0.05) but no significant impact on self-reliance (*B* = 0.02, SE = 0.37, *t*(96) = 0.06, *p* > 0.05). For specific details, refer to Figure 2.

The direct effect of group on self-reliance was significant (effect size = 2.66, Bootstrap 95% CI: [0.897, 4.417]), while the indirect effect via autonomy needs was not significant (effect size = 0.03, Bootstrap 95% CI: [−1.247, 1.343]). The total effect was significant (effect size = 2.69, Bootstrap 95% CI: [1.442, 3.941]), indicating that the mediation model for self-reliance was not supported.

The direct effect of group on self-assertion was not significant (effect size = −0.51, Bootstrap 95% CI: [−2.636, 1.623]), but the indirect effect via autonomy needs was significant (effect size = 2.20, Bootstrap 95% CI: [0.509, 3.985]). The total effect was also significant (effect size = 1.70, Bootstrap 95% CI: [0.118, 3.273]), suggesting that autonomy needs fully mediated the relationship between the Creative Dance intervention and self-assertion, thus validating the mediation model in this case.

The direct effect of group on self-control was not significant (effect size = 1.65, Bootstrap 95% CI: [−0.304, 3.603]), nor was the indirect effect via autonomy needs (effect size = 1.55, Bootstrap 95% CI: [−0.167, 3.433]). However, the total effect was significant (effect size = 3.20, Bootstrap 95% CI: [1.772, 4.617]), indicating that this mediation model was not valid (see Table 4).

Overall, these results demonstrate that satisfaction of autonomy needs significantly mediated the positive impact of the Creative Dance intervention on the children’s self-assertion. In contrast, the indirect effects of autonomy need satisfaction on self-reliance and self-control were not significant, suggesting that other mechanisms may explain the positive effects of Creative Dance in these domains. Further research is warranted to explore additional contributing factors.

## 5. Discussion

### 5.1. Creative Dance Promotes Young Children’s Autonomy Development

Based on a randomized controlled intervention experiment, this study systematically examined the effect of a Creative Dance intervention on the autonomy development of 4- to 6-year-old children. The findings confirmed the effectiveness of the intervention: following the 10-week program, the children in the experimental group (Creative Dance) showed significantly superior performance across all dimensions of autonomy (self-reliance, self-control, and self-assertion) compared to the control group (DanceSport), given the absence of significant baseline differences. This outcome robustly reflects the unique advantage of Creative Dance in fostering the multidimensional development of young children’s autonomy. Specifically, the analyses revealed differential sensitivity across the autonomy dimensions to the intervention: self-control (SC) exhibited the most pronounced improvement, evidenced by a strong time and group interaction effect (*F* = 19.87, *p* < 0.001). This significant advantage underscores that the core elements of Creative Dance, such as cooperative exploration and the requirement for precise body movement control, exert a powerful influence on enhancing children’s behavioral regulation and inhibitory control. Self-reliance (SR) also demonstrated a highly significant positive effect (*F* = 18.28, *p* < 0.001). This finding suggests that the intervention effectively cultivated the children’s ability to rely on their own resources for task completion, primarily through structured opportunities for independent choreography and improvisational expression. Self-assertion (SA) showed a statistically significant improvement (*F* = 4.55, *p* < 0.05). This enhancement likely stemmed from the curriculum’s focus on encouraging the children to engage in proactive decision-making during physical expression and collaborative dance communication, thereby reinforcing their initiative and articulation of personal intent. Collectively, these findings are highly consistent with the relevant theories and empirical studies on autonomy development, emphasizing the synergistic development process of autonomy as a multidimensional ability (Cuypers, 2010).

It is worth noting that the Creative Dance intervention significantly enhanced the children’s self-assertion and self-reliance and effectively promoted self-control development. According to the Self-Determination Theory, when individuals are in an environment that allows full autonomous exploration, they are more likely to internalize external norms as part of their self-worth, thereby generating self-disciplined behaviors driven by intrinsic motivation (Wang et al., 2019). Furthermore, the “autonomous exploration” and “improvisational dance” in Creative Dance are not equivalent to “unrestricted exploration” or “uninhibited dance”; during the activities, the children still needed to follow specific rules. However, these rules were not externally imposed constraints but structural resources that support autonomous exploration. This consistency between “rules and autonomy” lays a psychological foundation for improving self-control (Bonneville-Roussy et al., 2013). The following is an activity segment and analysis from the sixth week of the intervention experiment in the Creative Dance experimental group. The theme was “Changing Weather” and the learning elements were “flow” and “balance”. It can help us more directly understand how Creative Dance teaching promotes children’s internalization of rules and improvement of self-assertion by building a framework for their autonomous exploration:

*In the Creative Dance class themed “Changing Weather,” the teacher first introduced movement elements such as “flow” (freedom and constraint) and “balance” (stability and transcending balance). After the children had fully experienced and mastered these movement characteristics, the teacher set a clear exploration framework for the whole class: each child, in different weather scenarios (e.g., “rainy day” and “sunny day”), could give full play to their individual imagination and sensibility, freely expressing their understanding and emotions through body movements, with no expression being right or wrong. At the same time, the teacher emphasized that during the dance, they should try to use the movement elements learned that day, maintain a safe distance from peers, and immediately freeze in their current posture when they hear the music stop. As the music melody changed, the children all entered the scenario. Some imitated the light rotation of the body with their arms outstretched like falling drizzle while others tried to use high jumps to show the brightness and vitality of a sunny day. The teacher stood by, guiding and encouraging gently. For example, they praised a child who used arm circles to represent “wind blowing through tree branches”—this affirmed the child’s creativity and inadvertently inspired their peers to make more diverse attempts. In addition, when two children accidentally got too close, the teacher promptly reminded them softly, “Remember to keep a safe distance!” and invited them to think about adjusting their direction. In this process, the children gradually learned to be self-aware and adjust, rather than relying on external instructions. When the music stopped, all the children spontaneously froze in their current movements. Some children stood steadily with a smile while others struggled to adjust their center of gravity to prevent falling. Even those who were usually active and restless learned to wait and control their actions through the repeated cycle of “stop–move–stop again.” After class, some children communicated with each other, saying things like “I almost bumped into you just now, but I turned around to avoid it” or “How did you think of using such big arm movements to draw the sun?” These spontaneous interactions promoted emotional connections among peers and provided rich real-life materials for mutual learning and imitation. This classroom atmosphere of “rule–autonomy transformation” not only ensured the safety and order of the activity but also effectively stimulated the children’s intrinsic motivation and enthusiasm for active participation. The teacher flexibly balanced structure and freedom, enabling the children to dare to try while also learning to restrain themselves and respect and tolerate others. In the process of autonomous exploration and improvisational expression, the children gained experiences of subjectivity and a sense of achievement; at the same time, in abiding by and internalizing rules, they acquired self-discipline, a sense of responsibility, and enhanced self-control.*

This form of activity also contrasts with the aforementioned overly child-centered educational activities. While such activities provide ample freedom, the lack of explicit structural and rule-based scaffolding makes it difficult for young children to develop stable self-regulation mechanisms, which may hinder the development of self-control (Zhong, 1995; Cassidy, 2023). In contrast, Creative Dance operates within a scaffolded teaching framework, where teachers set appropriate rules and boundaries based on movement elements and exploration themes. These rules are flexible and contextually adaptable, dynamically adjusting according to the children’s understanding and ability levels, enabling them to gradually comprehend the rules’ rationality through continuous meaning-making processes. In contexts such as dance games and improvisational movements, children transition from passively following rules to actively constructing and upholding them. This transformation reflects the moral development process of transitioning “from heteronomy to autonomy”: when individuals internalize rules and view rule-following as a voluntary choice, self-control abilities become internalized and stably maintained (Song, 2022). From a psychological perspective, appropriate rules provide a sense of security and belonging, enhancing social cognition and collective responsibility. When rules are autonomously accepted, individuals are more likely to exhibit sustained and effective self-regulation in group settings (Zheng, 2006). In summary, by granting children sufficient autonomy and scaffolded space for independent exploration, Creative Dance can promote children’s acceptance of rules, thereby facilitating the transformation to self-discipline. This explains why Creative Dance strengthens children’s self-assertion and self-reliance in expression and action and effectively enhances their self-control abilities with structured support.

### 5.2. Factors Contributing to the Effectiveness of Creative Dance Intervention

This study not only further validates the role of creative dance in promoting autonomy among Chinese preschool children but also achieves specific innovations in intervention design, content selection, and teaching methods. We believe these innovations may be key factors contributing to the positive outcomes of this research, specifically manifested in: First, this study employs movement elements grounded in Laban theory as its primary instructional content. Building upon this foundation, it integrates the four core elements—Body, Effort, Shape, and Space (BESS)—with engaging themes favored by young children. This breaks away from the traditional “imitation-based” dance teaching model, creating a fun and expansive space for children to express their creativity independently (Engdahl et al., 2024). For example, in themed activities such as “Animal Carnival,” the children must independently select and combine movement elements around the theme and engage in continuous creative expression through dance exploration, group communication, role-playing, and improvisation. This design provides children with sufficient autonomous choice and expression space and enhances their active participation and aesthetic experience. Second, the scaffolded teaching approach was adopted. Appropriate prompts and guidance were given when the children encountered difficulties or confusion, but the overall initiative was left to the children. The teaching process focused on the “theme–question–dance exploration–sharing–internalization” sequence, enabling the children to think independently, discuss collaboratively, and actively adjust. This is conducive to the internal generation and consolidation of autonomy. Third, this type of activity involves improvisational dance, group collaboration, and giving presentations. Improvisational dance encourages children to make unique movements in the moment based on their imagination and strengthens self-identity through discussion and evaluation; group collaboration/presentation allows children to experience negotiation, self-expression, and self-regulation, comprehensively improving their multidimensional autonomy abilities in various scenarios.

### 5.3. The Unique Role of Autonomy Needs in the Process of Creative Dance Promoting Young Children’s Self-Assertion

Further analysis of the mediation effects revealed that satisfaction of autonomy needs significantly mediated the relationship between the Creative Dance intervention and self-assertion, whereas no significant mediation was found for self-reliance or self-control. This discrepancy may be attributable to the differential sensitivity of the various facets of autonomy to psychological need satisfaction. According to the Self-Determination Theory, whether an individual’s behavior is autonomous depends on whether they perceive autonomy in choice and the activation of intrinsic motivation. The theory posits that when autonomy needs are satisfied, intrinsic motivation is enhanced and behavioral internalization is promoted, allowing individuals to more effectively maintain self-assertion in the face of external influences (Ryan & Deci, 2020). These findings are consistent with the results of the present study. Specifically, Creative Dance provides young children with diverse autonomous choices (such as task selection, movement selection, and partner selection), encourages autonomous expression (dance performances and movement innovation), and offers positive feedback and peer support. These designs fully satisfy young children’s autonomy needs. The satisfaction process promotes a positive cycle of “I can–I want–I dare,” making children less likely to follow others’ opinions unthinkingly, enabling them to express unique insights and creative achievements, thus promoting self-assertion.

Mechanistically, there are three possible explanatory paths: First, the teaching context of Creative Dance stimulates autonomous experience through the right to choose and express, which can enhance young children’s participation motivation and the development of self-assertion. Second, through teacher support and peer interaction, young children internalize external norms and peer feedback into their self-regulations, supporting their positions in various situations. Finally, the internalized self-regulations become a driving force for young children to maintain self-assertion when facing external pressures (Black & Deci, 2000). In other words, Creative Dance does not simply allow children to “dance freely.” A structured exploration framework, autonomous choice rights, and supportive responses promote a continuous process of experiencing to understanding, internalization, and behavioral expression in young children. Meanwhile, the “movement–cognition” synergistic development path: Movement and physical expression are not passive displays of actions but important aspects for constructing subjectivity and identity (James, 2000). The improvisational dance and presentation sessions provided by Creative Dance allow young children to experience the feelings of “I can do it and I can be seen” through physical movements, thereby promoting self-assertion. Furthermore, Laban’s movement elements (BESS) provide a multidimensional medium of expression in the classroom, enabling young children to construct individualized movement vocabularies across four dimensions: Body, Effort, Shape, and Space. This integration of movement and cognitive expression further facilitates the development of self-assertion (Theodotou, 2025).

## 6. Limitations and Prospects

This study provides valuable empirical reference for understanding how Creative Dance promotes young children’s autonomy development and the role of autonomy needs in this process, enriching the practical knowledge in related fields. However, it still has certain limitations. First, the intervention period of this study was relatively short, and the sample size was limited (10 weeks and 102 participants) so the long-term stability and generalizability of the results need further verification. Future studies can be conducted with larger and cross-regional groups of young children, combined with long-term follow-up measurements, to explore the effectiveness of Creative Dance in different age groups and cultural contexts. Second, the intervention was mainly implemented in kindergarten without fully considering the impact of different environmental aspects, such as family and community, on autonomy development. According to ecological systems theory, family parenting styles, the quality of peer interaction, social culture, and other factors may significantly affect intervention outcomes (Bronfenbrenner, 1979). In the future, home–kindergarten collaboration programs, such as parent training and parent–child Creative Dance activities, can be designed to promote the transfer and stable development of children’s autonomy in different contexts.

Third, although this study found that Creative Dance can effectively promote self-reliance, self-control, and self-assertion, there were still differences in the development levels of these three dimensions among different children, indicating the importance of individual differences in interventions. Future research can incorporate variables such as the children’s temperaments, emotional regulation abilities, previous movement experience, and dance experience to explore differentiated and precise intervention paths, thereby enhancing the adaptability and targeting of Creative Dance interventions. Fourth, the assessment of the young children’s autonomy in this study utilized parent-proxy reports. In early childhood research, parent-proxy reporting is a commonly used approach that balances reliability, validity, and practical feasibility, providing stable and comprehensive data for evaluating young children’s autonomous behaviors. However, this approach may also introduce bias as parents’ perceptions are susceptible to social desirability effects (a tendency to report positive outcomes) and are inherently constrained by the frequency of their observations, which may not fully capture children’s autonomous behavior within the structured preschool environment. Future research should integrate multi-informant, multi-method assessment strategies, including direct observations of children’s behavior during the Creative Dance intervention, teacher reports, and age-appropriate child self-reports (where feasible), to obtain a more objective and comprehensive picture of autonomy development.

Building upon the findings of this study, a promising direction for future research lies in examining the translational potential of Creative Dance’s positive impact on child autonomy in the domain of ’aesthetic sports’—such as figure skating, rhythmic gymnastics, and artistic swimming—where artistic expression and creativity are paramount. Specific avenues include investigating the efficacy of implementing Creative Dance as a foundational preparatory curriculum, and developing innovative pedagogical models that embed autonomy-supportive strategies within technical skill acquisition. Such endeavors would not only extend the theoretical purview of autonomy theory into early-stage athletic development but also yield innovative frameworks for cultivating athletes who achieve synergy between technical excellence and artistically expressive depth.

In summary, Creative Dance has great potential in promoting young children’s autonomy development, and the satisfaction of autonomy needs plays a unique role in mediating its effects. However, further research is needed with larger samples, more contexts, and more refined strategies to construct a scientific, systematic, and promotable theory and practice system for cultivating young children’s autonomy based on Creative Dance.

## 7. Conclusions

By comparing the effects of Creative Dance and DanceSport on young children’s autonomy, this study found that Creative Dance significantly improved young children’s self-reliance, self-assertion, and self-control, with outcomes superior to those observed in the DanceSport group. The mediation analysis revealed that the satisfaction of autonomy needs played a significant mediating role in the relationship between the Creative Dance intervention and the development of self-assertion in the children. This indicates that developing young children’s self-assertion using Creative Dance depends on satisfying their autonomy needs. However, in the two dimensions of self-reliance and self-control, the satisfaction of autonomy needs did not show a mediating effect. This result suggests that the mechanism through which Creative Dance promotes these two dimensions may be more complex, and further exploration is needed in combination with exploration of other psychological factors or external factors. Our results emphasize the potential of Creative Dance in supporting young children’s autonomy needs and promoting the development of autonomy. Future research should further examine the causal mechanism of Creative Dance interventions and the optimal duration of intervention, as well as systematically evaluating its long-term benefits. In summary, incorporating Creative Dance into early childhood education intervention programs could help improve young children’s autonomy and respond to their actual needs through early childhood education, providing practical and effective references for the all-around development of young children and improving education quality.

## Figures and Tables

**Table 1:** Homogeneity test of pre-intervention self-reliance, self-assertion, and self-control levels between the two groups of young children (N = 102).

Variable	Group	N	M ± SD	*t*	*p*
Self-Reliance	Experimental	52	22.17 ± 3.78	−0.63	0.532
	Control	50	22.64 ± 3.75		
Self-Assertion	Experimental	52	22.08 ± 3.61	0.06	0.954
	Control	50	22.04 ± 2.84		
Self-Control	Experimental	52	28.63 ± 3.81	1.59	0.114
	Control	50	27.56 ± 2.62		

**Table 2:** Analysis of impact of teaching experiments on variance in young children’s levels of self-reliance, self-assertion, and self-control.

Variable	Comparison Type	Group/Time	Pre-Test M ± SD	Post-Test M ± SD	*F*	*p*	*η* ^2^
SR	Within-group comparison	EG	22.17 ± 3.78	25.75 ± 2.30	63.06	<0.05	0.39
		CG	22.64 ± 3.75	23.34 ± 3.05	3.15	0.079	0.03
	Main effect of time				10.90	<0.001	0.10
	Main effect of group				3.53	0.063	0.04
	Time × group interaction				18.28	<0.001	0.16
SA	Within-group comparison	EG	22.08 ± 3.61	24.67 ± 2.75	23.28	<0.001	0.19
		CG	22.04 ± 2.84	23.10 ± 2.64	3.00	0.087	0.03
	Main effect of time				0.23	0.63	0.002
	Main effect of group				3.30	0.07	0.33
	Time × group interaction				4.55	<0.05	0.05
SC	Within-group comparison	EG	28.63 ± 3.81	33.29 ± 2.91	86.37	<0.001	0.47
		CG	27.56 ± 2.62	29.00 ± 4.20	8.09	<0.01	0.08
	Main effect of time				1.38	0.243	0.01
	Main effect of group				24.52	<0.001	0.20
	Time × group interaction				19.87	<0.001	0.17

Note: SA = self-assertion; SC = self-control; SR = self-reliance; EG = experimental group; CG = control group.

**Table 3:** Correlation analysis of variables (*n* = 102).

Variable	Group	Self-Reliance	Self-Assertion	Self-Control
Group	1			
Self-Reliance	−0.411 ***	1		
Self-Assertion	−0.191	0.076	1	
Self-Control	−0.415 ***	0.241 *	0.319 **	1
Autonomy Needs	−0.691 ***	0.282 **	0.346 **	0.441 **

Note: * = *p* < 0.05; ** = *p* < 0.01; *** = *p* < 0.001.

**Table 4:** Mediation model effects.

Variable		Effect Size	BootSE	Bootstrap 95% CI Lower Bound	Bootstrap 95% CI Upper Bound
SA	Direct effect	2.66	0.887	0.897	4.417
	Indirect effect	0.03	0.658	−1.247	1.343
	Total effect	2.69	0.630	1.442	3.941
SR	Direct effect	−0.51	1.073	−2.636	1.623
	Indirect effect	2.20	0.875	0.509	3.985
	Total effect	1.70	0.795	0.118	3.273
SC	Direct effect	1.65	0.984	−0.304	3.603
	Indirect effect	1.55	0.893	−0.167	3.433
	Total effect	3.20	0.717	1.772	4.617

**Figure A1:**
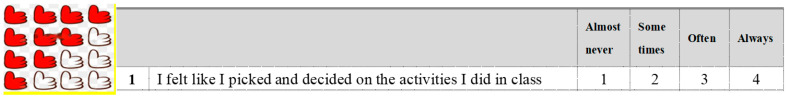
The Need for Autonomy Scale.

## Data Availability

The raw data supporting the conclusions of this article will be made available by the authors on request.

## Appendix A. Intervention Program

### Appendix A.1. Creative Dance

**Table A1:** Program of Creative Dance Intervention.

Week	Theme	Movement Elements	Course Objectives
Week 1	Intelligent Robot	Body parts; Levels: High, Medium, Low	Recognize and control different body parts; explore spatial levels of different heights, cultivate young children’s ability to complete movements independently, and enhance body-spatial coordination and independent exploration abilities.
Week 2	Bubbles and Jellyfish	Size: Large, Medium, Small; Energy: Sustained, Sudden	Experience changes in body movements in terms of size and energy; enable children to independently choose movement size and energy expression, modify rhythm and energy, and attempt independent creation of movement combinations, thereby fostering creativity and expressiveness in movements.
Week 3	Swimming Fish	Path: Straight line, Zigzag line, Wavy line; Speed: Fast, Slow; Position: Personal/General space	Explore diverse movement paths and speed variations; allow children to independently select movement paths and speeds to develop spatial awareness, direction control ability, as well as self-assertion and self-control.
Week 4	Underwater World	Body shapes: Curved, Round, etc.; Body relationships: Above, Below, Separate, Follow, Guide, etc.	Enrich physical expression; encourage children to independently design movement combinations for interaction with peers, cultivating cooperation, following, guiding, and self-reliance.
Week 5	Fruit Battle	Body shapes: Needle-shaped, Wall-shaped, Spherical, Spiral, Symmetrical, Asymmetrical	Stimulate creative body shaping; enable children to independently select shape combinations and symmetrical forms, enhancing spatial awareness and creativity.
Week 6	Changing Weather	Flow: Free, Restricted; Balance: Balanced, Beyond balance	Improve the stability and controllability of movements; encourage children to attempt different balance challenges and independently adjust movement strategies, strengthening responsiveness and self-control.
Week 7	Story of the Four Seasons	Flow: Free, Restricted; Energy: Sustained, Sudden; Speed: Fast, Slow; Rhythm: 2/4, 4/4 (rhythm patterns)	Integrate rhythm, speed, and energy elements; cultivate children’s ability to combine and create movements with different speeds and rhythms, enhancing musicality and sense of rhythm in movements and promoting the development of self-assertion.
Week 8	Animals Gather for a Meeting	Weight: Light, Heavy; Body relationships: Individual vs. Others, Mirroring	Enhance children’s perception of weight and strength; through mirror imitation and interactive sessions, help children develop cooperation, imitation, and self-control abilities.
Week 9	Animal Carnival	Integration of learning content, Dance drama creation	Apply the learned movement elements to Creative Dance choreography; encourage children to independently design dance segments and conduct team collaboration, promoting the development of collective creativity and autonomy.
Week 10	Animal Carnival	Integration of learning content, Dance drama creation	Complete and present the dance drama work; enable children to independently participate in performance choreography and role assignment, consolidating creativity and collective cohesion and promoting the development of autonomy.

### Appendix A.2. DanceSport

**Table A2:** Program of DanceSport Intervention.

Week	Course Content	Course Objectives
Weeks 1–2	Basic Latin dance standing posture, Latin dance hand positions; Cha-cha on-the-spot hip rotation, Cha-cha timing steps	Establish correct dance posture and body coordination and master the basic rhythm of Cha-cha and the key points of hip movements.
Weeks 3–4	Cha-cha timing steps, Cha-cha box steps; Hand position combinations	Improve movement accuracy and sense of rhythm and enhance upper limb coordination and dance expressiveness.
Weeks 5–6	Cha-cha New York steps; Partner practice	Strengthen rapport and interaction with peers and improve spatial perception and collaborative abilities.
Weeks 7–8	Basic Cha-cha group combinations	Comprehensively apply the learned steps and hand positions to enhance dance fluency and expressiveness.
Weeks 9–10	Integrated practice; Performance rehearsal	Consolidate movement memory and dance expression, and complete preparations for stage presentation.

## Appendix B. Research Materials

### Appendix B.1. Autonomy of Children

Instructions: We will continue to learn about your child’s behavioral characteristics through the following items. Each is a single-choice question, and after each sentence, there are five possible answers: 1 = completely inconsistent, 2 = slightly inconsistent, 3 = generally consistent, 4 = mostly consistent, and 5 = completely consistent. Please read each sentence carefully and decide which answer is closest to your child’s actual situation, then put a “√” on the corresponding number. Please answer every question, even if you are unsure; just choose the answer that seems closest. Please do not spend too long on any one question.

**Table A3:** Children’s Autonomy Scale.

		Completely Inconsistent	Slightly Inconsistent	Generally Consistent	Mostly Consistent	Completely Consistent
1	Washes small items such as handkerchiefs or socks by themselves	1	2	3	4	5
2	Can put on long pants independently	1	2	3	4	5
3	Squeezes toothpaste and brushes teeth by themselves	1	2	3	4	5
4	Can bathe themselves with simple help (applying soap, scrubbing, wiping, etc.)	1	2	3	4	5
5	Can skillfully use chopsticks	1	2	3	4	5
6	Returns to calm quickly after their request is refused by parents	1	2	3	4	5
7	Parents wash the child’s face in the morning	1	2	3	4	5
8	Verbally agrees when asked to do something by parents, but does not take action	1	2	3	4	5
9	Does not leave the seat during meals	1	2	3	4	5
10	Can state the reasons for others’ goodness or badness	1	2	3	4	5
11	Can clearly express requests to parents	1	2	3	4	5
12	Can express own opinions when talking with parents	1	2	3	4	5
13	Can completely report tasks given by kindergarten teachers to parents	1	2	3	4	5
14	Knows the reason for liking cartoon characters	1	2	3	4	5
15	Has own ideas when doing things	1	2	3	4	5
16	Lies on the ground and cries when throwing a tantrum	1	2	3	4	5
17	Can share favorite food with friends	1	2	3	4	5
18	Listens quietly to peers without interrupting others	1	2	3	4	5
19	Stops own activity immediately when being stopped	1	2	3	4	5
20	Goes to bed at the designated time at home	1	2	3	4	5

### Appendix B.2. The Need for Autonomy Scale (Interview)

Guideline: Children, next your teacher will ask you questions separately; there are no right or wrong answers, just like painting, and there is no best answer, only your favorite. Just choose the answer you feel most right according to your own feeling. Each question is followed by four numbers, like four little steps. If it feels that the questions are exactly the same as your usual, choose step 4; if you feel that a lot of it is the same as your usual, choose step 3; if it is just a little bit like your usual, choose step 2; if you think it is super different from your usual, choose step 1. Take your time to think about it and choose the answer you think it is.

Examples: picking/deciding (e.g., letting me choose my own dance style, choosing classmates to dance with, deciding what moves I want to do, etc.).

### Appendix B.3. Observation of Autonomous Need-Supportive Teaching Behaviors

Autonomy-supportive teaching behaviors contain three items. Furthermore, need-support behaviors were coded every 5 min using a 4-point frequency scale ranging from (never observed) to 1 (sometimes observed) to 2 (often observed) and 3 (always observed).

**Table A4:** Observation of Autonomous Need-Supportive Teaching Behaviors.

Physical Education Teachers’ Need-Supportive Observation Point		Time	0–5	5–10	10–15	15–20	20–25	25–30	30–35	35–40
		Rating	0–3	0–3	0–3	0–3	0–3	0–3	0–3	0–3
1	Offers choice to the students (for example, the choice of practice sequence, the choice of teaming up; in the course, students can choose the practice method, students can choose the classmates they want to cooperate with)	Autonomy Support								
2	Offers the opportunity to experience problems, to practice independently, to experiment, to exercise and to solve problems on their own, without interfering	Autonomy Support								
3	Differentiated instruction (for example, teachers provide exercises of varying difficulty, considering the possibilities for different students (groups))	Autonomy Support								
